# Cigarette Smoke Induced Airway Inflammation Is Independent of NF-κB Signalling

**DOI:** 10.1371/journal.pone.0054128

**Published:** 2013-01-22

**Authors:** Joseph M. D. Rastrick, Christopher S. Stevenson, Suffwan Eltom, Megan Grace, Meirion Davies, Iain Kilty, Steven M. Evans, Manolis Pasparakis, Matthew C. Catley, Toby Lawrence, Ian M. Adcock, Maria G. Belvisi, Mark A. Birrell

**Affiliations:** 1 Respiratory Pharmacology, National Heart and Lung Institute, Faculty of Medicine, Imperial College London, London, United Kingdom; 2 Roche, Nutley, New Jersey, United States of America; 3 Pfizer, Pfizer Inc, Cambridge, Massachusetts, United States of America; 4 University of Cologne, Cologne, Germany; 5 AstraZeneca, Molndal, Sweden, United Kingdom; 6 Centre d'Immunologie Marseille-Luminy, Marseille, France; 7 Airway Disease, Imperial College London, United Kingdom; St. Jude Children's Research Hospital, United States of America

## Abstract

**Rationale:**

COPD is an inflammatory lung disease largely associated with exposure to cigarette smoke (CS). The mechanism by which CS leads to the pathogenesis of COPD is currently unclear; it is known however that many of the inflammatory mediators present in the COPD lung can be produced via the actions of the transcription factor Nuclear Factor-kappaB (NF-κB) and its upstream signalling kinase, Inhibitor of κB kinase-2 (IKK-2). Therefore the NF-κB/IKK-2 signalling pathway may represent a therapeutic target to attenuate the inflammation associated with COPD.

**Aim:**

To use a range of assays, genetically modified animals and pharmacological tools to determine the role of NF-κB in CS-induced airway inflammation.

**Methods:**

NF-κB pathway activation was measured in pre-clinical models of CS-induced airway inflammation and in human lung tissue from COPD patients. This data was complemented by employing mice missing a functional NF-κB pathway in specific cell types (epithelial and myeloid cells) and with systemic inhibitors of IKK-2.

**Results:**

We showed in an airway inflammation model known to be NF-κB-dependent that the NF-κB pathway activity assays and modulators were functional in the mouse lung. Then, using the same methods, we demonstrated that the NF-κB pathway appears not to play an important role in the inflammation observed after exposure to CS. Furthermore, assaying human lung tissue revealed that in the clinical samples there was also no increase in NF-κB pathway activation in the COPD lung, suggesting that our pre-clinical data is translational to human disease.

**Conclusions:**

In this study we present compelling evidence that the IKK-2/NF-κB signalling pathway does not play a prominent role in the inflammatory response to CS exposure and that this pathway may not be important in COPD pathogenesis.

## Introduction

Chronic obstructive pulmonary disease (COPD) is an inflammatory lung disease characterised by a progressive decline in lung function and a largely irreversible airflow obstruction. It is typically associated with cigarette smoke (CS) exposure and as the prevalence of COPD continues to rise, its financial and medical burden to society continues to grow [1]. Indeed it is predicted that by 2030 COPD will become the third largest cause of death worldwide [2], however treatment options are limited and there are currently no drugs available which can stop the progressive course of this disease [3].

Current dogma suggests that the persistent particulate/oxidative burden caused by smoking can generate secondary mediators such as cytokines, growth factors and proteases that are responsible for the structural and functional changes seen in the COPD lung (such as emphysema, narrowing of the small airways and airflow limitation). These mediators are also thought to be responsible for the infiltration of inflammatory cells recruited to the COPD lung including neutrophils, macrophages and lymphocytes [4], [5] in particular CD8^+^ T cells and B cells [6], [7]. Although the exact mechanisms driving this cellular infiltration and subsequently the pathogenesis of COPD remain unclear, literature suggests that the genes transcribing many of these pro-inflammatory mediators are regulated by the transcription factor Nuclear Factor-κB (NF-κB) [8], [9]. Consistent with this view there is some evidence to support a role for the NF-κB pathway in the pathogenesis of COPD [10], [11].

The aim of this study was to perform a comprehensive assessment of the role of the NF-κB pathway in the inflammatory response associated with CS exposure and the development of COPD. To do this we adopted 3 approaches; firstly to measure the level of NF-κB pathway activation in the airways of CS driven pre-clinical models of COPD and in lung samples from diseased patients. Secondly, to profile 2 strains of genetically modified mice missing functional IKK-2, a key NF-κB signalling kinase in purported COPD effector cells (specifically, in airway epithelial cells (IKK-2^ΔEpi^) or in cells of myeloid lineage (IKK-2^ΔMye^)) in the model systems. Finally, as total IKK-2 KOs are embryonically lethal [12] we used systemic pharmacological inhibitors of IKK-2, (TPCA-1, [13], [14] and GSK 657311A, [15]), to globally inhibit the pathway. To validate the NF-κB assays and the various tools described above, we employed a parallel model system of aerosolised lipopolysaccharide (LPS)-induced airway inflammation. The bacterial endotoxin LPS has previously been reported to activate the NF-κB signalling pathway through the Toll-Like Receptor (TLR)-4 [16] and we have previously shown inflammation in a similar model system to be NF-κB-dependent and sensitive to IKK-2 inhibition [17].

## Methods

### Animals

Male C57BL/6 mice (18–20 g) were obtained from Harlan UK Limited (Bicester, UK) and housed for at least 5 days before beginning treatments with food and water supplied *ad libitum*. Conditional knockout (KO) mice lacking IKK-2 in the airway epithelium were provided by Pfizer (Sandwich, UK) and were originally generated in the laboratory of Professor Pasparakis (University of Cologne, Germany) and were a donation from Pfizer (Sandwich, UK). Mice lacking IKK-2 specifically in cells of myeloid lineage were donated by Dr. Toby Lawrence (Centre d'Immunologie Marseille-Luminy, France) and were originally generated in the laboratory of Michael Karin (University of California, USA). Both strains of conditional KO mice were on a C57BL/6 background and GM/wild type controls were either bred by Charles River UK (IKK-2^ΔEpi^) or in house (IKK-2^ΔMye^). All protocols were approved by a local ethical review process and strictly adhered to the Animals (Scientific Procedures) Act 1986 UK Home Office guidelines.

To generate the IKK-2 targeted gene deletion, a mouse line expressing Cre-recombinase (Cre) was crossed with a mouse line expressing the IKK-2 gene, flanked (or *floxed*) either side with inserted *loxP* sites (IKK-2^f/f^). In these mice the Cre-recombinase gene is targeted to a specific cell type via the Surfactant protein-C (SP-C) gene exclusive to lung epithelial cells [18] or the Lysozyme-M (Lys-M) gene exclusive to myeloid cells [19]. Cre-recombinase is a site-specific DNA recombinase that catalyses the recombination of DNA between *loxP* sites. Therefore the *floxed* IKK-2 gene will only be excised from cells expressing Cre-recombinase. Furthermore the IKK-2^ΔEpi^ mice also express a reverse tetracycline transactivator (rtTA) in order to induce the conditional KO with doxycycline. IKK-2^ΔEpi^ mice were administered doxycycline in the drinking water on embryonic day 14 during early lung morphogenesis allowing Cre/loxP recombination to occur throughout the airway epithelium under the control of the SP-C promoter [18], [20]. By selectively targeting IKK-2 in these cell types the survival rate of the mice is not affected compared to wild type C57BL/6 mice and no phenotypical differences are observed. In order to control for the technology used to generate the IKK-2^ΔEpi^ mice, age and sex-matched control groups included wild type C57BL/6 mice, IKK-2^f/f^ mice, IKK-2^f/f^ mice expressing rtTA only (IKK-2^f/f^rtTA^+^) and IKK-2^f/f^ mice expressing Cre recombinase only (IKK-2^f/f^Cre^+^). Control groups for the IKK-2^ΔMye^ mice included wild type C57BL/6 mice and IKK-2^f/f^ mice.

Greten *et al* [21] and Takahashi *et al* [22] have shown that this line of IKK-2^ΔMye^ mice have approximately a 75% deletion efficiency of IKK-2 in bone-marrow-derived macrophages (BMDM) and neutrophils and 80–90% deletion in alveolar macrophages. There are currently no publications that have analysed the deletion efficiency of IKK-2 in these IKK-2^ΔEpi^ mice, however Perl *et al* [18] have demonstrated that Cre recombinase induced by doxycyline under the control of the SP-C promoter is expressed in virtually all respiratory epithelial cells.

### Models

Temporal characterisation of the LPS and CS-induced models of airway inflammation were performed as described in Eltom *et al*, [23]. Briefly, LPS challenging was performed in a Perspex chamber (600×240×350 mm) using a System 22 nebuliser (Medic-Aid Ltd., Pagham, Sussex) driven by a high-flow-rate compressor (Medic-Aid Ltd., Pagham, Sussex). Animals were exposed to either aerosolised LPS (sub-maximal dose of 1 mg/ml [23], *Escherichia coli*, serotype 0111:B4, Sigma-Aldrich Ltd. Poole, UK) or endotoxin free saline (Fresenius Kabi, Warrington, UK) blown into the Perspex chamber for a 30 minute challenge period. CS was generated from filterless 3R4F cigarettes (Tobacco Health Research Institute, University of Kentucky, Lexington, KY) drawn through an exposure chamber (136L capacity, Teague Enterprises, CA, USA) by negative pressure using an extraction pump (Grainger Industrial Supply, USA). The flow-rate was set at 1500 ml/min and a timed pinch-valve (C. Lee Machining, Horsham, UK) was used to draw CS through the chamber for 2 seconds every 4 seconds. This generates a final flow through the chamber of 500 ml/min. By adjusting the flow rate and pinch valve timings the concentration of cigarette smoke within the chamber was controlled. Between draws of cigarette smoke, room air was continuously drawn through the chamber. To ensure an even and continuous dispersal of cigarette smoke, a fan was placed at the base of the chamber and Total Smoke Particulate (TSP) levels were determined every 15 minutes using a TSP sampling unit (Teague Enterprises, CA, USA). Each exposure period lasted for 50 minutes followed by a 10 minute venting period at maximum flow. The sub-maximal dose of smoke (500 ml/min) was selected from dose response experiments previously published by this group [23].

### Temporal characterisation

The LPS challenge model consisted of a single challenge, whereas the CS driven model had two challenge protocols. The first protocol was two challenges per day (4 hours apart) for three days (minimum number of challenges required for a robust airway neutrophilic response, [23]), the second challenge protocol was chosen on data published by our group recently [23] showing that after 14 days of exposure the phenotype of inflammation changes to include an increase in airway macrophages and lymphocytes.

To generate samples to assess inflammatory status and compare it to the activity status of the NF-κB pathway, animals were culled at increasing time points (2, 6, 24, 48, 72, 96 and 168 hours) after the final challenge with an overdose of i.p sodium pentobarbitone (200 mg/kg). Bronchoalvelolar lavage fluid (BALF) was collected by tracheal cannulation and instillation of the lungs with 0.3 ml of Roswell Park Memorial Institute 1640 medium + GlutaMAX-I (RPMI, Invitrogen, Paisley, UK,) for 30 seconds. This was removed and repeated twice more, pooling the samples for each animal. Aliquots of each sample were then used for total leukocyte and differential cell counts. The differential cell counts were measured as a percentage of the total leukocyte count and were determined from cytocentrifuge preparations. These were prepared by centrifuging 100 ul BALF in a cytospin (Shandon, Runcorn, UK) at 700 rpm for 5 min at standard room temperature and pressure. The slides were then fixed and stained on a Hema-tek 2000 (Ames Co., Elkhart, IN, USA) using modified Wright-Giemsa stain (Sigma-Aldrich Ltd., Poole, UK, WG128). Differential cell counts were carried out on 200 cells per slide using standard morphological criteria under light microscopy. The remaining BALF was spun at 800×g and the supernatant retained at −80°C until required for cytokine measurement (by either MSD technology or using specific ELISAs from R&D systems, UK). The lung tissue was collected and flash frozen in liquid nitrogen.

### Detection of NF-κB:DNA association

To measure the activity status of the NF-κB pathway we used the TransAM^TM^ ELISA-based plate assay according to the manufacturer's instructions. Briefly, an oligonucleotide containing a consensus sequence for NF-κB (5′-GGGACTTTCC-3′) was immobilized to the bottom of each well in a 96-well plate. Nuclear protein was extracted from the lung tissue using a CelLytic NuCLEAR Extraction kit (Sigma Aldrich, UK) according to manufactures instructions. Total protein was assessed using a Bradford assay and then samples added to each well (10 ug protein/well) and incubated for 1 hour before the addition of primary antibodies specific for NF-4444B subunits (p65, p50, p52 or RelB). Each well was then incubated with a horseradish peroxidase-antibody conjugate for 1 hour and developed with 3,3′,5,5′-Tetramethylbenzidine (TMB) to from a blue colour product. The reaction was then stopped using an acidic stop solution and absorption was determined at 450 nm on a spectrophotometer (Biotek PowerWave XS Plate Reader, Potton, UK).

### Confirmation that the genetic alterations have impacted on the phenotype of the IKK-2^ΔEpi^ mice

As indicated above a great deal of information is known about the IKK-2^ΔMye^ mice, however there is less known about the IKK-2^ΔEpi^ mice. In order to demonstrate that IKK-2 was deficient in the airway epithelium of IKK-2^ΔEpi^ mice we used two methods, firstly we collected the epithelial cells from these mice and measured IKK-2 expression by RT-PCR and secondly, to show a function of the loss of IKK-2, LPS-induced p65 nuclear translocation in the lung was assessed by IHC.

### Primary mouse airway epithelial cell culture

To culture mouse airway epithelial cells, mice were euthanised with an overdose of i.p. sodium pentobarbitone (200 mg/kg) and sprayed with 70% ethanol. Tracheas were then aseptically removed from the tracheal bifurcation to the larynx and cleared of all connective tissue. The trachea was then washed twice in PBS and incubated for 1 hour at 37°C in 1 mg/ml dispase. The tissue was then washed in Small Airway Growth Media with Bullet kit supplements (SAGM Bullet kit, Lonza, Cologne, Germany, CC-3118) and epithelial cells were lightly scraped from the trachea into a collagen-coated 30 mm Petri dish. The cells were cultured in Small Airway Growth Media with supplements until approximately 80% confluent.

### RNA extraction from primary mouse airway epithelial cells

RNA was isolated from airway epithelial cells using Tri Reagent (Sigma, Poole, UK). Briefly, the cells were washed in PBS and resuspended in 1 ml Tri Reagent. The upper aqueous phase was collected according to the manufacturer's instructions and centrifuged with 200 ul chloroform in nuclease-free centrifuge tubes. The aqueous fraction was mixed with 1/10 its volume of isopropanol to precipitate the RNA which was then washed in 70% (v/v) ethanol. The RNA pellet was left to air dry for no more than 10 minutes and resuspended in 50 ul nuclease-free water. The purity and concentration of RNA was then quantified using the ratio of absorptions at 260/280 nm by spectrophotometry (Biotek PowerWave XS Plate Reader, Potton, UK).

RNA (0.5 ng/ml) samples were reverse transcribed using a master mix containing 1x Taqman buffer, 5.5 mM MgCl_2_, deoxynucleotide triphosphate (dNTP) mix (500 µM per NTP), 2.5 mM Random hexamers, 0.4 U/ml RNase inhibitor and 1.25 U/ml multiscribe reverse transcriptase in a final reaction volume of 50 ul. The reaction mix was incubated in a Perkin Elmer 480 thermal cycler (Perkin Elmer, Boston MA, USA) for 10 minutes at 25°C, reverse transcribed at 48°C for 30 minutes and finally the enzyme was denatured at 95°C for 5 minutes to yield approximately 10 ng/ul cDNA.

### IKK-2 mRNA expression in primary mouse airway epithelial cells

Transcriptional expression of IKK-2 mRNA transcripts were analysed in airway epithelial cells using TaqMan real-time quantitative polymerase chain reaction (PCR). Fluorescent-labelled TaqMan probes were designed to amplify the floxed exons of the IKK-2 gene. These probes were designed to target exons 6 and 7 and were purchased from Applied Biosystems. These floxed exons should be absent in cells expressing Cre-recombinase.

PCR reactions were performed using a master mix containing 2x TaqMan universal master mix, MgCl_2_, random dNTPs, the specific fluorescent-labelled Taqman probe for each conditional knockout and sample cDNA (10 ng/ul) in a final reaction volume of 25 ul. Amplification and detection of the specific products was carried out using the ABI PRISM 7000 Sequence Detection System (Applied Biosystems, Warrington, Cheshire, U.K.) with an amplification protocol of 1 cycle at 50°C for 2 minutes, 1 cycle at 95°C for 10 minutes, 40 cycles at 95°C for 15 seconds, and 1 minute at 60°C.

Each PCR was carried out in single-plex and the basic eukaryotic cell component 18 S was used as a positive control. The Taqman probes for 18 S used VIC as the reporter fluorophore and the IKK-2 probes used FAM (Applied Biosystems, Warrington, Cheshire, U.K.).

### Immunohistochemical staining of NF-κB(p65) in IKK-2^ΔEpi^ mice

In order to demonstrate a function of the loss of IKK2, we measured p65 translocation in IHC section from the lung after LPS stimulation. Briefly, 2 hours post LPS challenge; lungs were insufflated by tracheal cannulation with 10% neutral buffered formalin at a pressure of 20 mm Hg. The tissue was incubated in the fixative overnight after which the lungs were cleared and processed into paraffin blocks. Paraffin sections (3 μM) were then cut and mounted on to glass microscopy slides.

Mounted sections were de-waxed by immersion in Histoclear for 5 minutes. The Histoclear wax solvent was removed by immersing the sections twice in 100% ethanol for 5 min and once in 70% ethanol for 5 minutes. Alcohol was then washed from the sections with running tap water and the sections were transferred to 1% H_2_O_2_ in methanol for 10 minutes for peroxide quenching. Antigen retrieval was achieved by microwaving the sections for 10 minutes in EDTA (pH 8.0). Following this, the sections were incubated for 1 hour in Vectastain ABC blocking serum (Vectastain Universal-Anti-mouse IgG/ Rabbit IgG, Elite ABC kit, PK-6200, Vector Laboratories, Burlingame, Ca), for 15 minutes in Vectastain Avidin Blocker and for 15 minutes in Vectastain Biotin blocker (Vector Laboratories, Avidin/Biotin blocking kit, SP-2001), with 2×2 minutes washes between each blocking step (0.1% Triton, 0.1% BSA in PBS). The primary p65 antibody (sc-372, Santa Cruz, Santa Cruz, Ca) was then added to the sections (1∶500) and incubated for 1 hour. This was removed with 3×5 min washes and the sections were incubated with a secondary biotinylated antibody (Vector Laboratories, BA1400) for 30 minutes. The sections were washed again and incubated for 30 minutes in Vectastain Elite ABC reagent (Vector Laboratories, Vectastain universal, Anti-mouse IgG/ Rabbit IgG, Elite ABC kit, PK-6200). Finally the sections were stained using DAB peroxidase substrate solution (Vector Laboratories, SK-4100) until the desired stain intensity developed. The sections were counter-stained with haematoxylin and cover-slipped.

Slides were scored using an arbitrary scale of 0–4 denoting the intensity of NF-κB (p65)-DAB staining in airway epithelial cells. Sections were scored over 10 random fields/ section at ×40 magnification (0 =  no stain, 4 =  intense brown stain) by 2 independent scorers blinded as to the origin of the samples.

### The role of epithelial cell IKK-2 in LPS-induced airway inflammation

Having confirmed that the genetic manipulation in the IKK-2^ΔEpi^ mice had reduced levels of IKK2, we wanted to demonstrate that in a proof of concept model known to be NF-κB-dependent that these mice had an altered inflammatory status. Wild type and genetically altered mice were exposed to aerosolised saline or LPS and samples collected 2 (for p65 TransAM assessment) or 6 hours (for cytokines and cellular inflammation) later.

### The role of epithelial cell IKK-2 in CS-induced airway inflammation

Having used the NF-κB-dependent LPS model to demonstrate a functional effect of the IKK-2^ΔEpi^ mice they were used as a testing tool in our 3 and 14 day smoke models. Wild type and genetically altered mice were exposed to air or CS for 3 or 14 days. Samples were collected 24 hours after the last challenge and assessed for cytokines and cellular inflammation.

### The role of myeloid cell IKK-2 in LPS-induced airway inflammation

Wild type and genetically altered mice were exposed to aerosolised saline or LPS and samples collected 2 (for p65 TransAM assessment) or 6 hours (for cytokines and cellular inflammation) later.

### The role of myeloid cell IKK-2 in CS-induced airway inflammation

Having used the NF-κB dependent LPS model to demonstrate a functional effect of the IKK-2^ΔMye^ mice we profiled their response to 3 and 14 day CS exposure. Wild type and genetically altered mice were exposed to air or CS for 3 or 14 days. Samples were collected 24 hours after the last challenge and assessed for cytokines and cellular inflammation.

### The role of IKK-2 in LPS-induced airway inflammation

To test if IKK-2 in cell types other than epithelial cells or myeloid cells (or a combination of all cell types) could be involved in the CS models we turned to pharmacological inhibitors. As with the genetically modified mice, we first wanted to establish the effectiveness of the pharmacological tools in the LPS-driven model. We profiled two structurally different IKK-2 inhibitors (TPCA1 and GSK 657311A) and a clinically relevant glucocortoid, budesonide. Compounds were orally dosed 60 minutes prior to LPS challenge and samples collected 2 (for p65 TransAM assessment) or 6 hours (for cytokines and cellular inflammation) later.

### The role of IKK-2 in CS-induced airway inflammation

Having selected doses using the NF-κB dependent LPS model we profiled them in the 3 day smoke model. Mice were dosed 60 minutes prior to the morning CS challenge and 60 minutes after the afternoon CS challenge. Samples were collected 24 hours after the last challenge and assessed for cytokines and cellular inflammation.

### Human tissue

Human lung nuclear extracts were generated from lung tissue taken from non-smoking donors, healthy smoking donors and emphysema patients (demographic details are published [23]). Informed written consent was obtained for the use of human tissues for research and ethical approval from the Royal Brompton and Harefield ethics committee was obtained (09/H0708/72). NF-κB pathway activity was determined by measuring NF-κB(p65): DNA association in the nuclear fraction of tissue extracts using a TransAM plate assay (Active Motif) according to the manufacturer's instructions.

### Statistical analysis

Data is expressed as mean ± s.e.m of n observations. Statistical significance was determined using either Student's t-test or One-way ANOVA/Kruskal-Wallis followed by an appropriate post-hoc test depending on whether the data was parametric or non-parametric. A P value <0.05 was taken as statistically significant and all treatments were compared with appropriate control groups.

## Results

### Temporal characterisation of LPS-induced airway inflammation

**Figure 1:**
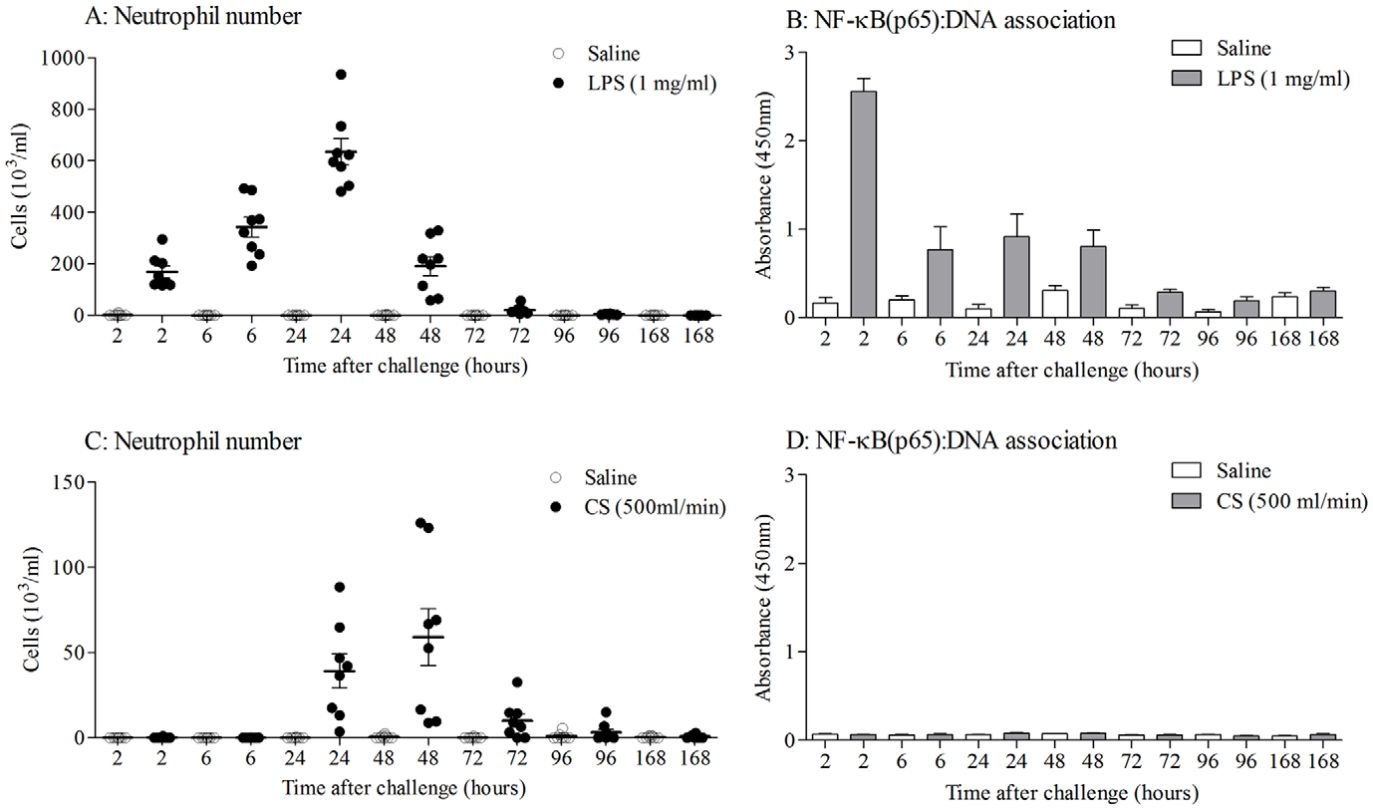
Temporal characterisation of the airway inflammation after LPS or 3 days of CS challenge. Mice were challenged with LPS (1 mg/ml) or endotoxin free saline for 30 minutes. Samples were collected at increasing time points after challenge. A) LPS-induced BALF neutrophilia. B) LPS-induced NF-κB(p65):DNA association in lung nuclear extract. Mice were challenged for 3 days with CS (500 ml/min, 1 hour, twice daily) or ambient air. Samples were collected at increasing time points after the final challenge. C) CS-induced BALF neutrophilia. D) CS-induced NF-κB (p65):DNA association in lung nuclear extract. Data are presented as mean ± s.e.m. of n = 6–8 observations.

Aerosolised LPS resulted in an increase in BALF leukocyte numbers, predominantly consisting of neutrophils (Figure 1A). Nuclear extracts from lung tissue homogenates had higher levels of NF-κB (p65): DNA association after LPS challenge compared with vehicle time matched controls, indicating an increase in NF-κB pathway activation (Figure 1B). This was temporally associated with an increase in cytokine levels such as Keratinocyte-derived chemokine (KC), Tumour Necrosis Factor (TNF)α and interleukin (IL)-6, (Figure S1).

### Temporal characterisation of 3 day CS-induced airway inflammation

**Figure 2:**
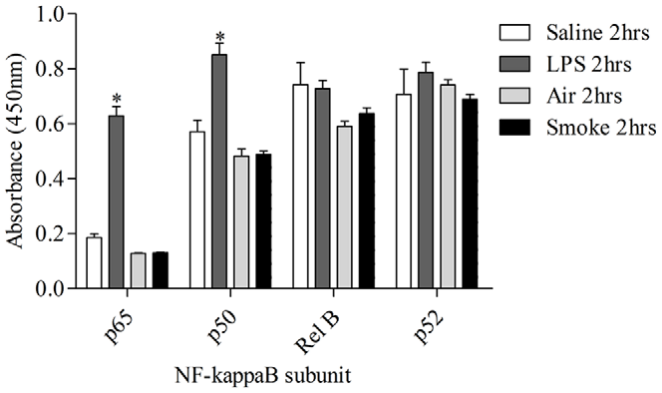
Characterisation of NF-κB pathway activation after LPS or 3 days of CS challenge. Mice were challenged with LPS (1 mg/ml) or endotoxin free saline for 30 minutes or challenged for 3 days with CS (500 ml/min, 1 hour, twice daily) or ambient air. Samples were collected 2 hours after the last challenge. NF-κB subunits were measured in the lung nuclear fractions. Data are presented as mean ± s.e.m. of n = 6–8 observations. * indicates statistical significance (p<0.05) against saline control group (Students t test).

**Figure 3:**
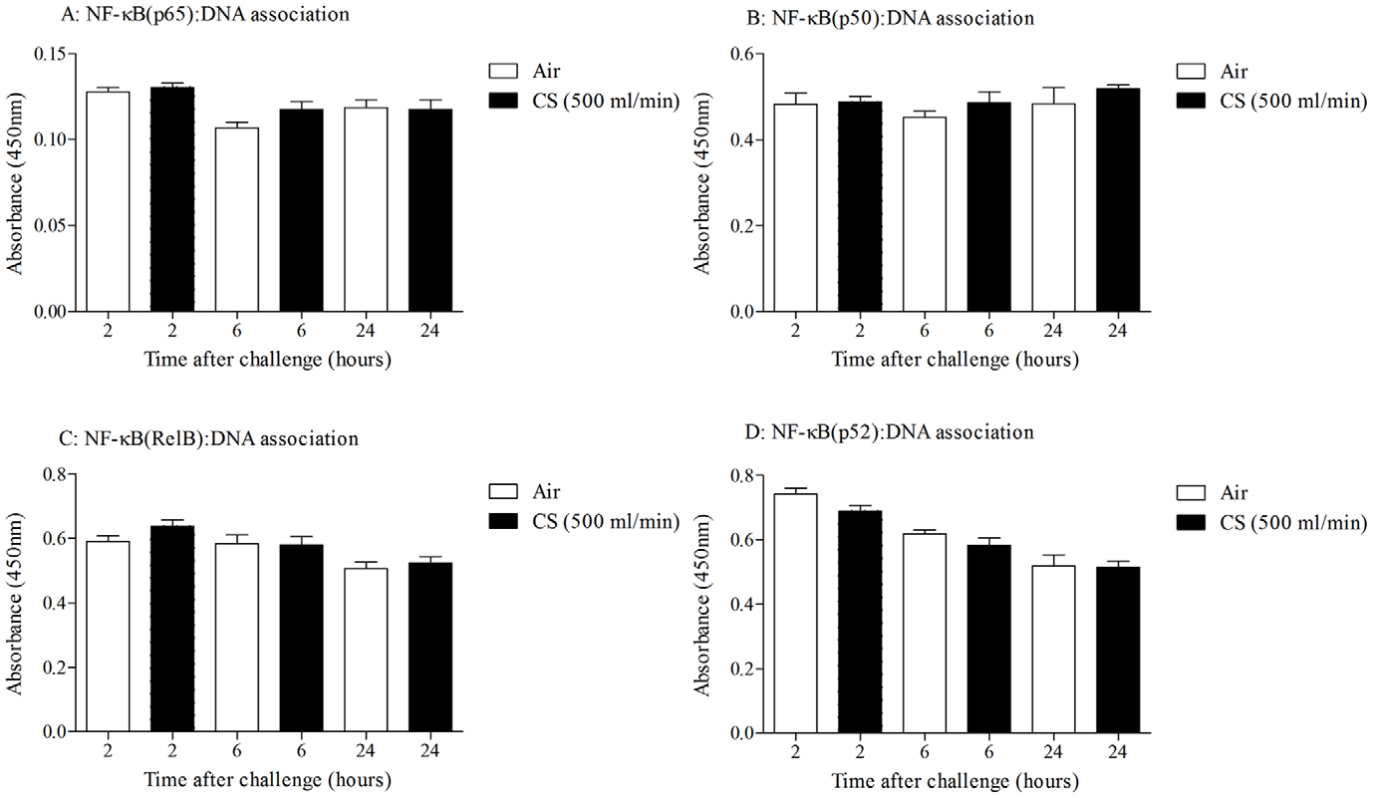
Temporal characterisation of NF-κB pathway activation after 3 days of CS challenge. Mice were challenged for 3 days with CS (500 ml/min, 1 hour, twice daily) or ambient air. Samples were collected at increasing times after the last challenge. NF-κB subunits were measured in the lung nuclear fractions. A: p65, B: p50, C RelB and D: p52. Data are presented as mean ± s.e.m. of n = 6–8 observations.

**Figure 4:**
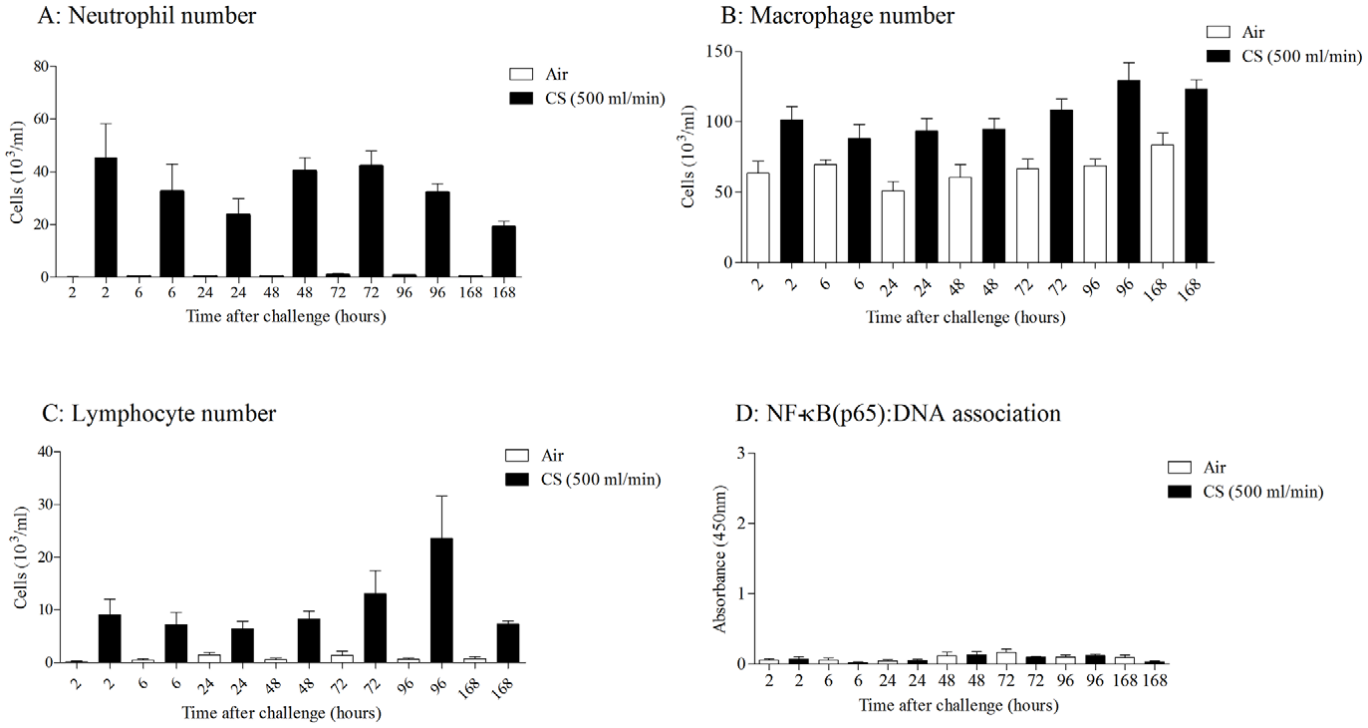
Temporal characterisation of airway inflammation after 14 days of CS challenge. Mice were challenged for 14 days with CS (500 ml/min, 1 hour, twice daily) or ambient air. Samples were collected at increasing time points after the final challenge. A) BALF neutrophilia, B) BALF macrophage number, C) BALF lymphocytes and D) NF-κB (p65):DNA association in lung nuclear extract. Data are presented as mean ± s.e.m. of n = 6–8 observations.

Exposure to 3 days of CS resulted in an increase in total BALF leukocyte number which was similar to LPS-induced airway inflammation and predominantly consisted of neutrophils (Figure 1C). This was temporally associated with an increase in cytokine levels such as KC, IL-23 (Figure S1), IL-1β and IL-18 [23]. In contrast to LPS however, CS-induced neutrophilia was not associated with an increase in NF-κB (p65): DNA association (Figure 1D). In order to determine whether other NF-κB family members played a role in CS-induced airway inflammation, DNA association with the NF-κB subunits p50, p52 and RelB were also determined in the lung tissue nuclear extracts. An increase in DNA association with the NF-κB family members p65 and p50, but not RelB and p52, was detected 2 hours after LPS challenge (Figure 2). However, we could not detect any changes in DNA association with the NF-κB family subunits following CS exposure (Figure 3 and 4). The NF-κB subunit c-Rel could not be determined in mice using the Active Motif TransAM assay.

### Temporal characterisation of 14 day CS-induced airway inflammation

As described earlier, the phenotype of cellular inflammation changes following continued exposure to CS to include increased numbers of macrophages and lymphocytes. Therefore we wanted to determine if there was any activation of NF-κB in this sub-chronic model. As can be seen from Figure 4 longer CS exposure led to an increase in neutrophils, macrophages and lymphocytes, and interestingly unlike the 3 day CS model the inflammation does not resolve over the same time scale. This cellular inflammation was temporally associated with an increase in cytokine levels such as KC and IL-23 (Figure S1). Yet, despite this marked inflammation we could not measure any increases in NF-κB pathway activation (Figure 4D).

### Validation of conditional epithelial IKK-2 KO mice

**Figure 5:**
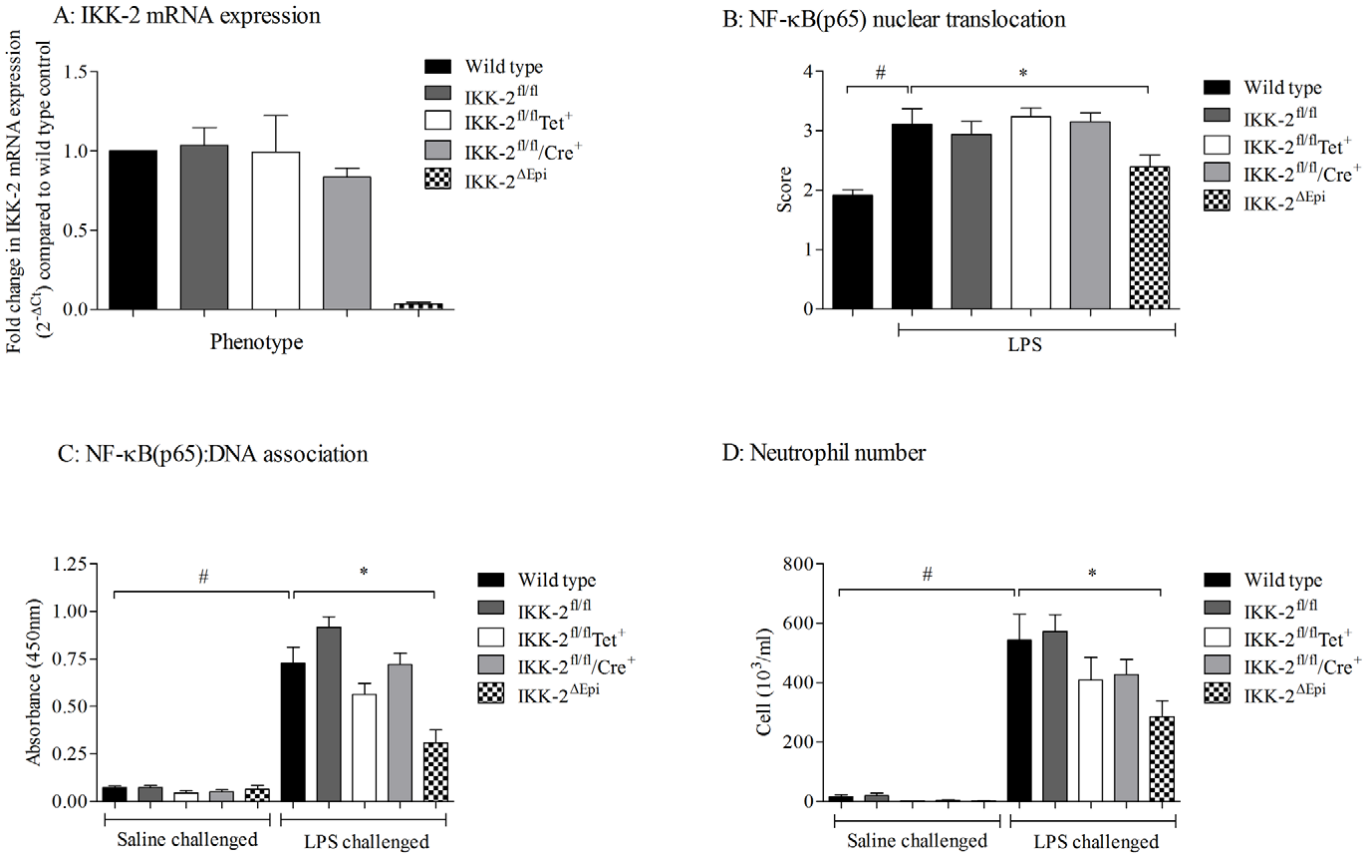
Characterisation of the IKK-2^ΔEpi^ mice. To demonstrate the knockdown of IKK-2 in the airway epithelium, mouse primary epithelial cells were harvested and cultured from control or IKK-2^ΔEpi^ mice and mRNA levels were determined by RT-PCR. Data is expressed as fold change from wild type control values (A). To demonstrate a function on the knockdown, mice were challenged with 1 mg/ml aerosolised LPS or saline and 2 hours after challenge lung tissue was collected for immunohistochemistry. Levels of p65 in the nuclei were assessed by 2 people blinded to the treatment groups. The score represents the mean ± s.e.m of 10 random fields performed by both scorers (B). To show this knockdown can impact on whole tissue extracts, NF-κB (p65):DNA association in lung nuclear extracts were measured (C). Finally to show the effect of disrupting the NF-κB signalling pathway in epithelial cells, mice were challenged with LPS and BALF was collected 6 hours later. Neutrophilia was measured in the BALF (D). # indicates a statistically significant difference (p<0.05) from saline challenged control groups (Mann-Whitney test). * indicates statistical significance (p<0.05) from LPS treated control groups by Kruskal-Wallis one-way-ANOVA with Dunn's multiple comparison post-hoc test. Any changes in data collected from the GM control mice i.e. fl/fl, TET alone and CRE alone did not reach statistical significance when compared to the appropriate control group.

Although we did not detect an increase in NF- κB pathway activity it is possible that by using extracts from the whole lung tissue we may have missed the signal in discrete areas/cell types of the lung. The conditional IKK-2 KO mice enabled us to investigate the role of NF-κB signalling in specific cell types thought to play a prominent role in COPD i.e. airway epithelial cells and myeloid cells. Before using the epithelial conditional IKK-2 KO mice in the CS exposure models we wanted to validate the knock down of IKK-2. To do this we collected primary mouse airway epithelial cells from the IKK-2 KO mice and measured IKK-2 mRNA expression. Figure 5A shows that there is a clear reduction in IKK2 mRNA expression in airway epithelial cells from IKK-2^ΔEpi^ mice compared to control phenotypes (Wild type C57/BL6, IKK-2^f/f^, IKK-2^f/f^rtTA^+^, IKK-2^f/f^Cre^+^. To demonstrate a function of this reduction in IKK2 we used IHC staining for NF-κB(p65) after LPS challenge and showed a reduction in nuclear staining in the epithelial cells of the IKK-2^ΔEpi^ mice (Figure 5B).

Finally to demonstrate that these genetically modified mice can have an altered inflammatory profile we tested them in an LPS-induced model of lung inflammation known to involve the NF-κB signaling pathway. As can be seen in Figure 5C and D, mice with reduced IKK-2 in airway epithelial cells also had reduced whole lung NF-κB(p65):DNA association and airway neutrophilia compared to the wild type and transgenic controls. We also recorded a commensurate decrease in inflammatory cytokines such as KC and TNFα (Figure S2).

### The role of epithelial IKK-2/NF-κB signaling in CS-induced airway inflammation

**Figure 6:**
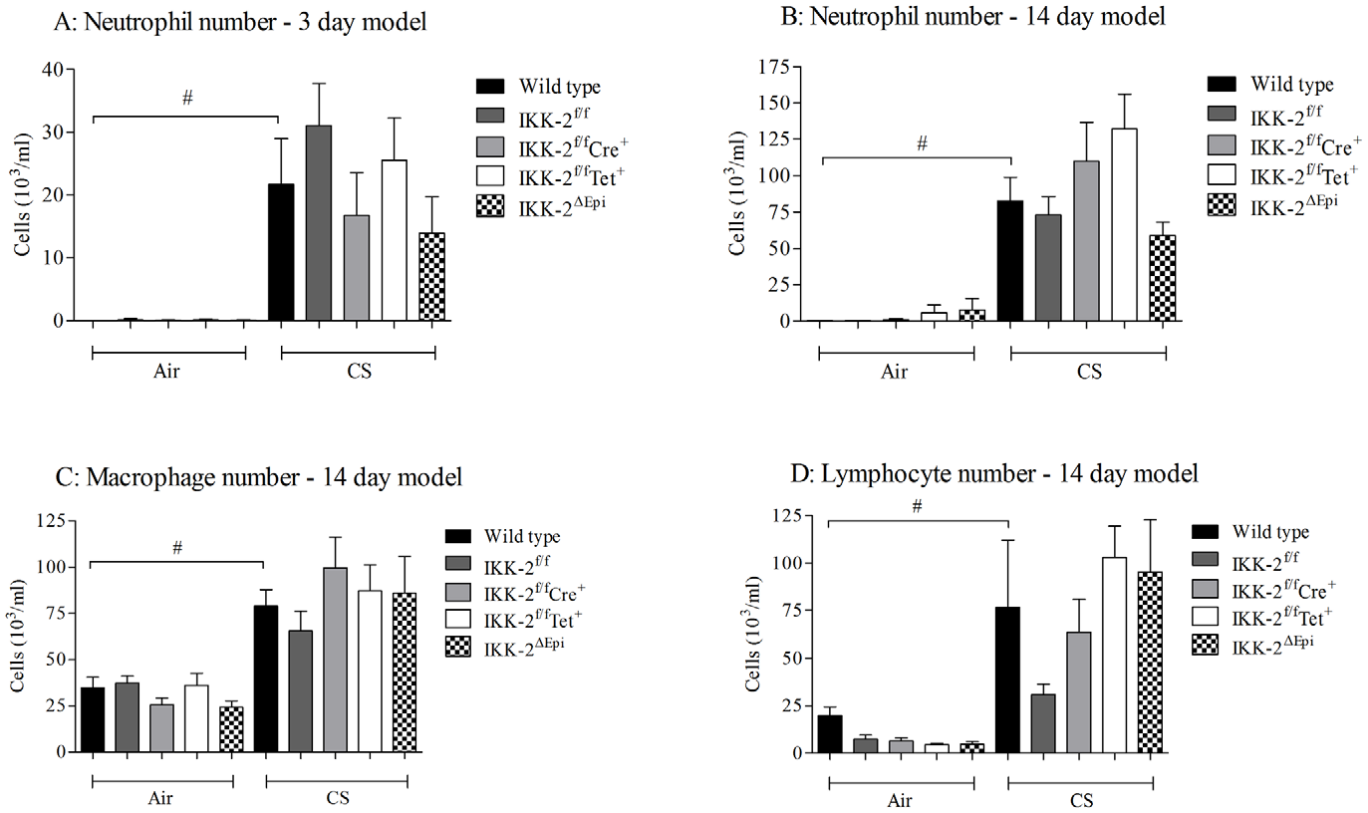
Profile of IKK-2^ΔEpi^ mice in the 3 and 14 day CS-driven models. IKK-2^ΔEpi^ mice were challenged with 3 or 14 days CS (500 ml/min, 1 hour, twice daily) or ambient air. BALF samples were collected twenty-four hours after the final challenge. BALF neutrophilia after 3 days of CS challenge (A) BALF neutrophilia, macrophages and lymphocytes after 14 days of CS challenge (B, C and D, respectively). Data are presented as mean ± s.e.m. of n = 8–16 observations. # indicates a statistically significant difference (p<0.05) from air challenged control groups (Mann-Whitney test). Any changes in data collected from the GM control mice i.e. fl/fl, TET alone and CRE alone did not reach statistical significance when compared to the appropriate control group.

Having confirmed that the IKK-2^ΔEpi^ mice had reduced levels of IKK2 and that this led to a functional reduction in NF-κB:DNA association, we characterized their response to 3 and 14 days of CS exposure. Acute CS exposure caused an increase in BALF neutrophil number 24 hours after challenge which was not significantly different in the IKK-2^ΔEpi^ mice (Figure 6B) when compared to the appropriate control groups. In parallel, CS-induced cytokines (KC and IL-1β) were also not significantly altered in the KO mice (Data not shown). In addition elevated BALF neutrophil, macrophage and lymphocyte numbers induced by 14 days of CS exposure were also not affected by ablation of IKK-2 in airway epithelial cells (Figure 6B–D). Again in parallel, CS induced cytokines (KC, IL-6, IL-23 and IL-1β) were not significantly altered in the KO mice (data not shown).

### The role of myeloid IKK-2/NF-κB signaling in CS-induced airway inflammation

**Figure 7:**
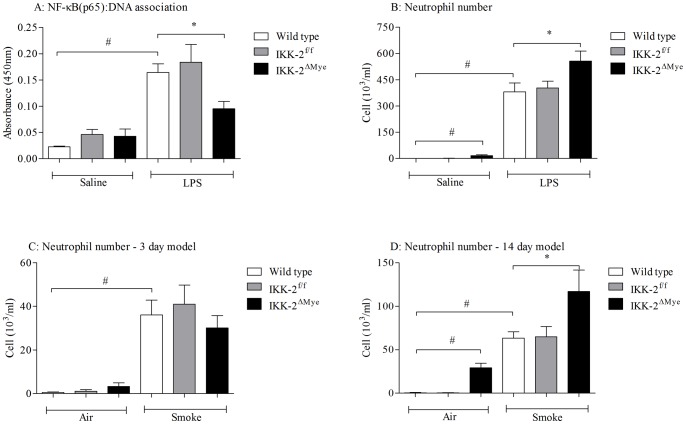
Characterisation of the IKK-2^ΔMye^ mice and inflammatory profile following 3 and 14 day CS exposure. IKK-2^ΔMye^ mice were challenged with LPS and lung tissue collected 2 hours later and BALF samples 6 hours later. Figure A represents the level of NF-κB pathway activation in the lungs and Figure B the BALF neutrophilia. IKK-2^ΔMye^ mice were challenged with CS for 3 or 14 days and BALF was collected twenty-four hours after the final challenge. Figures C and D represent the numbers of neutrophils in the BALF after 3 and 14 days, respectively. Data are presented as mean ± s.e.m. of n = 8 observations. # indicates a statistically significant difference (p<0.05) from saline/air challenged control groups (Mann-Whitney test). * indicates statistical significance (p<0.05) from LPS/CS treated control groups by Kruskal-Wallis one-way-ANOVA with Dunn's multiple comparison post-hoc test. Bars with no annotation were not significantly different from their respective control group.

As more work has been published on the IKK-2^ΔMye^ we felt we did not need to validate this strain to the same extent. We did however profile them in the LPS model of airway inflammation to demonstrate a functional response. Figure 7A shows that the IKK-2^ΔMye^ mice had a significant reduction in NF-κB pathway activation. Interestingly this was associated with an increase in BALF neutrophilia and associated cytokine release (Figure 7B and data not shown). When we characterized these mice in the 3 and 14 day CS exposure models there was no decrease in neutrophils and associated cytokines compared to wild type and transgenic control mice (Figure 7C, 7D and data not shown).

### Effect of IKK-2 inhibitor and budesonide on LPS/CS-induced airway inflammation

**Figure 8:**
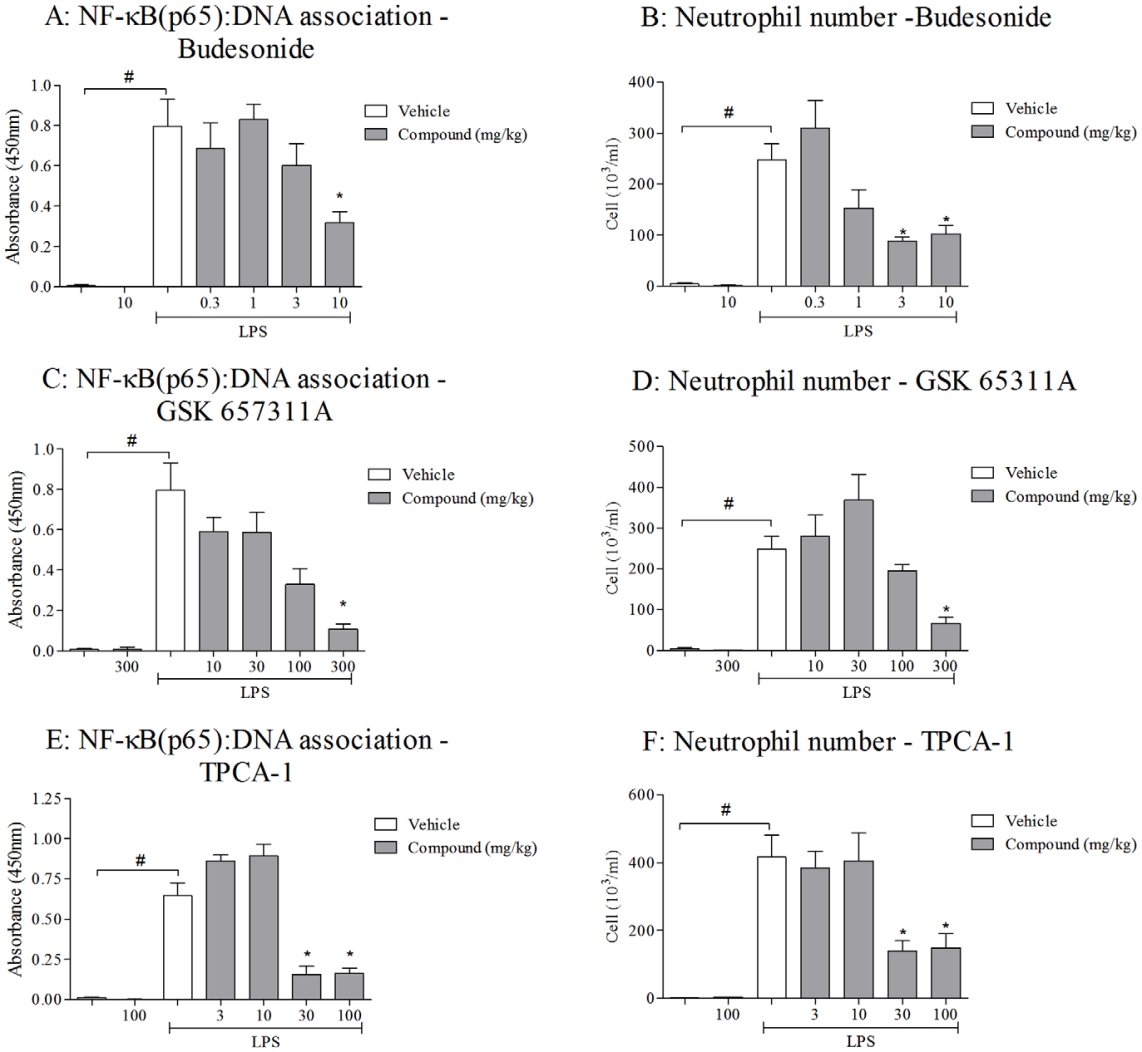
Characterisation of a clinically relevant glucocortoid, budesonide and two structurally distinct IKK-2 inhibitors GSK 657311A and TPCA-1 in LPS-induced airway inflammation. Vehicle or compound was orally dosed to the mice one hour prior to the LPS challenge. Lung tissue was collected 2 hours after LPS challenge and BALF samples 6 hours after challenge. Figures A, C and E represent the levels of NF-κB pathway activation in the lungs and Figure B, D and F the BALF neutrophilia. Data are presented as mean ± s.e.m. of n = 6–8 observations. # indicates a statistically significant difference (p<0.05) from control challenged groups (Mann-Whitney test). * indicates statistical significance (p<0.05) from LPS challenged vehicle dosed groups by Kruskal-Wallis one-way-ANOVA with Dunn's multiple comparison post-hoc test. Bars with no annotation were not significantly different from their respective control group.

To test if IKK-2 in cell types other than epithelial cells or myeloid cells (or a combination of all cell types) could be involved in the CS-induced models, we used pharmacological inhibitors of IKK-2 (TPCA-1 and GSK 657311A) and compared them with a clinically relevant glucocortoid, budesonide. Consistent with the ablation of IKK-2 in cells of myeloid lineage or in airway epithelial cells, the systemic inhibition of IKK-2 caused a significant decrease in NF-κB(p65): DNA association and BALF neutrophilia (Figure 8) following LPS exposure. We also recorded a commensurate decrease in inflammatory cytokines such as KC, TNFα and IL-6 (Figure S3). Budesonide also significantly decreased cytokines and neutrophilia and at the highest concentration reduced NF-κB(p65): DNA association (Figure 8 and Figure S3).

**Figure 9:**
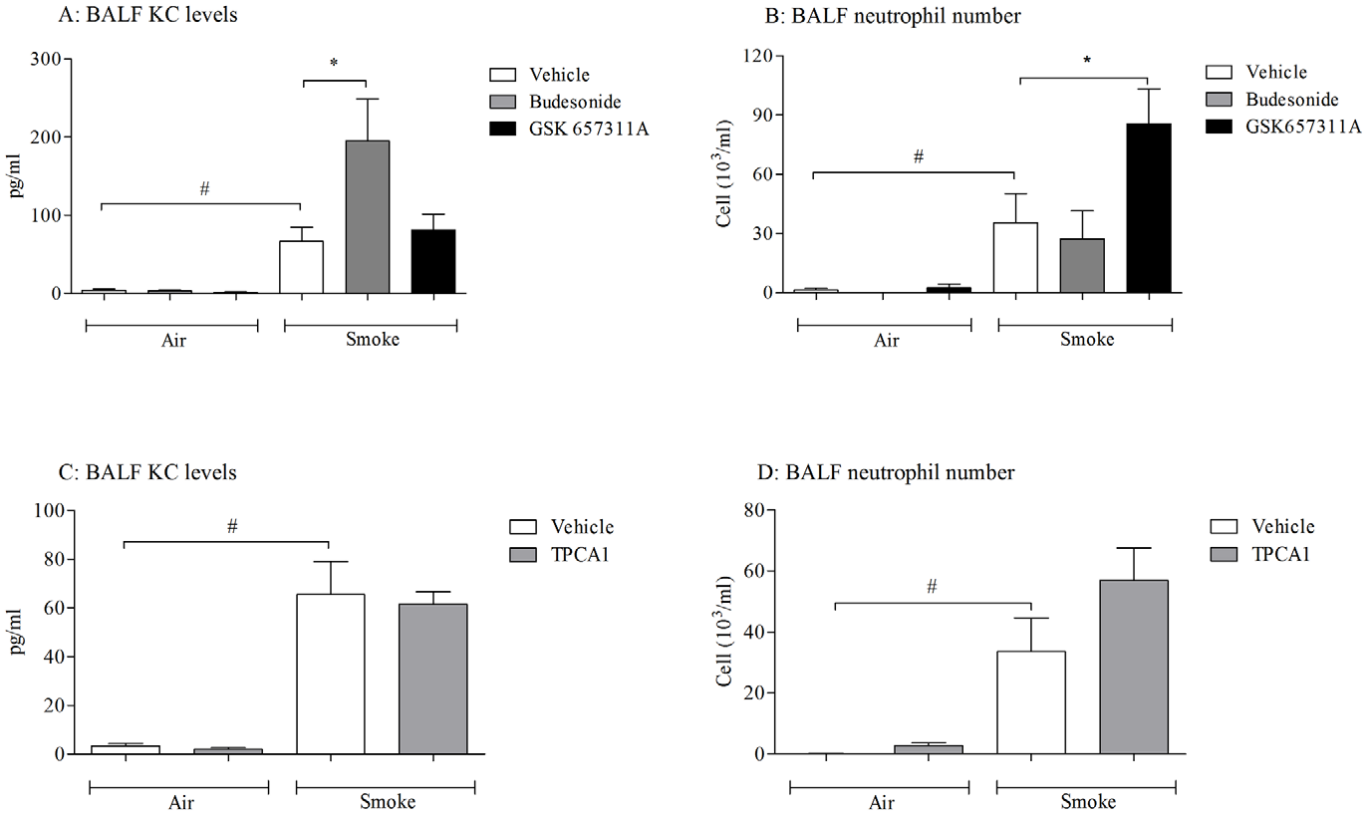
Profiling the impact of a clinically relevant glucocortoid, budesonide (3 mg/kg) and two structurally distinct IKK-2 inhibitors GSK 657311A (100 mg/kg) and TPCA-1 (30 mg/kg) in CS-induced airway inflammation. Vehicle or compound was orally dosed to the mice one hour prior to the morning CS challenge and one hour after the afternoon CS challenge. BALF samples were collected 20 hours after the last CS challenge. Figures A and C represent the levels of KC in the BALF and Figures B and D the BALF neutrophilia. Data are presented as mean ± s.e.m. of n = 6–8 observations. # indicates a statistically significant difference (p<0.05) from the air challenged control groups (Mann-Whitney test). * indicates statistical significance (p<0.05) from CS challenged vehicle dosed control groups by Kruskal-Wallis one-way-ANOVA with Dunn's multiple comparison post-hoc test. Bars with no annotation were not significantly different from their respective control group.

In contrast however, neither budesonide nor the inhibition of IKK-2 caused a reduction in CS-induced cytokine and neutrophil levels (Figure 9). Indeed budesonide significantly increased KC levels and IKK-2 inhibition tended to increase the neutrophilia. These findings are in support of the conditional knockout studies and suggest that NF-κB does not play a prominent role in CS-induced airway neutrophilia.

### NF-κB: DNA association in human tissue

**Figure 10:**
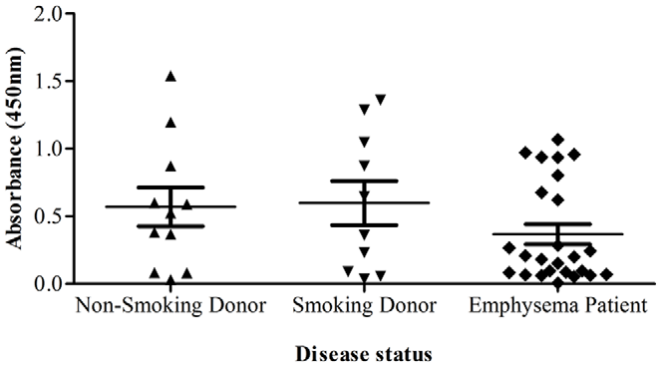
NF-κB:DNA association in human lung tissue. NF-κB(p65): DNA association in lung tissue from non-smoking donors, smoking donors and emphysema patients. Data are presented as mean ± s.e.m. of n = 10–25 observations. * indicates a statistically significant difference (p<0.05) from non-smoking donors by Kruskal-Wallis one-way-ANOVA with Dunn's multiple comparison post-hoc test.

In order to see if our observations from these pre-clinical models translated into human COPD we measured NF-κB(p65): DNA association in lung tissue from non-smoking donors, smoking donors and emphysema patients. In support of our pre-clinical models it can be seen that there is no significant difference in NF-κB(p65): DNA association between non-smoking donors, smoking donors and patients with emphysema (Figure 10).

## Discussion

The production of many of the pro-inflammatory mediators associated with the pathogenesis of COPD has been associated with the transcription factor NF-κB and as a result it has been suggested as a viable target for the treatment of COPD. Previous to this study, reports have suggested that there is enhanced NF-κB pathway activation in the lungs of COPD patients. Nuclear localisation of the NF-κB(p65) subunit has been recorded in sputum macrophages of COPD patients during exacerbations, but not in sputum neutrophils [10] and Di Stefano *et al* (2002) have also shown that the nuclear expression of NF-κB(p65) is increased in bronchial biopsies from COPD patients [11]. Several studies have also shown that CS exposure in preclinical models can cause an increase in NF-κB:DNA association in mice [24]–[29] and rats *in vivo* [30]–[32]. In addition Rajendrasozhan *et al* [33] observed that the pharmacological inhibition of IKK-2, attenuates acute CS-mediated BALF cytokine levels and neutrophilia. There is now however, an increasing amount of evidence to suggest that NF-κB pathway activation does not play a prominent role in COPD. A number of groups have shown that CS exposure has no effect on NF-4444B pathway activation in 129SvJ mice [25], [34] and Tharapel *et al* [35] reported that mice challenged with CS for up to 56 days showed no increase in NF-κB:DNA binding activity and at particular time points even showed a reduction. Indeed exposure to passive cigarette smoke is reported to inhibit UV-induced NF-κB(p65) signalling [36]. In addition to these murine models, CS has also been reported to inhibit IKK activity and NF-κB:DNA association in rats [37] and in Rhesus monkeys [38].

The role of NF-κB in patients with COPD is also controversial. Although NF-κB:DNA binding activity is reported to be increased in patients who smoke when compared to non-smoking patients, a difference in NF-κB pathway activity was not observed between “healthy” smokers and smokers with COPD [39]. Furthermore, Drost *et al* [40] reported a reduction in NF-κB:DNA binding activity in “healthy” smokers and patients undergoing severe COPD exacerbations compared to non-smoking individuals.

In this study we were unable to detect an increase in NF-κB:DNA association following both 3 day and 14 day CS exposure. In contrast to LPS–induced lung inflammation, acute CS-induced BALF neutrophilia was not associated with an increase in p65, p50, RelB or p52 DNA binding activity. When mice were exposed to CS for 14 days the cellular phenotypes detected in the BALF included elevated macrophage and lymphocyte numbers in addition to neutrophils which did not resolve even 7 days after the last challenge. Despite this increased and prolonged inflammatory response an increase in NF-κB(p65): DNA association was still not detected at any time point measured after challenge. It is possible that although extensive time points were investigated, the correct time point at which to detect a window of NF-κB pathway activation may have been missed. Another possibility is that NF-κB pathway activation could not be detected in a whole lung nuclear extract due to small localised increases in the lung generated by discrete cell types. We therefore utilised two strains of mice lacking functional IKK-2 in purported COPD effector cells; cells of myeloid lineage (IKK-2^ΔMye^) or the airway epithelium (IKK-2^ΔEpi^). These mice are phenotypically normal in contrast to mice with complete ablation of IKK-2 or NF-κB(p65) which results in fatal hepatocyte apoptosis during embryonic development [12], [41]. To confirm the genetic status of the less well characterised IKK-2^ΔEpi^ mice, we showed that IKK-2 mRNA expression in airway epithelial cells was dramatically reduced compared to cells from the control mice. A functional reduction in IKK-2/NF-κB signalling was also demonstrated as IKK-2^ΔEpi^ mice had reduced NF-κB(p65): DNA association following LPS challenge. In addition, IHC staining for NF-κB (p65) also showed an attenuation of nuclear translocation in targeted cells. When the genetically modified mice were exposed to 3 days or 14 days CS however, neither the IKK-2^ΔMye^ or IKK-2^ΔEpi^ mice showed any reduction in BALF neutrophilia or related cytokines. In fact, ablation of IKK-2 in cells of myeloid lineage actually caused an increase in BALF neutrophilia induced by 14 days CS. A possible explanation for this exaggerated inflammatory response has been provided by Greten *et al* [42] who report that myeloid specific IKK-2 deficiency is associated with an attenuation of neutrophil apoptosis and an increase in caspase-1 mediated IL-14444 processing. Alternatively Fong *et al* [43] have also reported that the deletion of IKK-2 in myeloid cells may be associated with an increase in STAT-1 transcriptional activation. Indeed, an NF-κB-independent role for IKK-2 has also been reported by others using small molecule IKK-2 inhibitors, including TPCA-1 [44]. This may help to explain why the inflammatory response to CS was also increased following the systemic inhibition of IKK-2 with TPCA-1 and GSK 657311A. It is possible that this effect is mediated through cells of myeloid lineage; however this remains to be determined. Another possibility is that the increased inflammatory response induced by the inhibition of IKK-2 may result in an increase in alternative NF-κB (RelB:p52) pathway activity mediated through IKK-1. Indeed it has previously been shown that p65 can suppress RelB:DNA association in the nucleus of fibroblasts [45]. However, as CS was unable to induce an increase in either classical (p65 and p50) or alternative (RelB and p52) NF-κB pathway activity it suggests that, in this model, neither pathway is dominant and suppressing the other.

Our data therefore suggests that contrary to other reports, IKK-2/NF-κB signalling in airway epithelial cells and in cells of myeloid lineage does not significantly contribute to CS-induced lung inflammation in these models. Previously Lee *et al* [46] demonstrated that ablation of IKK-2 in the airway epithelium via the Clara Cell (CC10) promoter significantly reduced CS-induced neutrophil and macrophage number in the BALF (no data on the level of NF-κB pathway activation was shown). It is not clear why our data differs from Lee *et al*; it could be differences in CS exposure protocol. A possible explanation could be that the concentration of smoke used in other models to induce inflammation could reduce oxygen levels to an extent that causes hypoxia, which is reported to induce NF-κB mediated gene transcription [47]. Another difference is the promoter used to generate the knockout. We utilised the surfactant protein-C (SP-C) promoter which is expressed early on during lung morphogenesis. By inducing the knockout with doxycyline at the correct time point during embryonic development, the SP-C promoter is reported to direct transgene expression in both the conducting airways and the alveolar epithelium [20] whereas IKK-2 deletion via the CC10 promoter is largely expressed in the airway epithelium but not in the alveolar epithelium [48].

To confirm our findings we also used two small molecular weight IKK-2 inhibitors TPCA-1 and GSK 657311A to globally inhibit IKK-2/NF-κB signalling. Treatment dose-dependently inhibited NF-κB(p65): DNA association in the lung, cytokine production and reduced BALF neutrophilia following LPS challenge. However in the CS driven model the compounds had no significant effect on BALF cytokines or neutrophilia. Furthermore, in contrast to LPS-induced lung inflammation, treatment with the corticosteroid budesonide also did not reduce CS-induced lung inflammation. This correlates with data indicating that steroids have a limited impact on inflammatory indices in COPD [49], [50]. As glucocorticoid treatment is believed to act in part by modulating the actions of NF-κB, this is more evidence that NF-κB signalling does not play a key role in CS-induced lung inflammation. Finally we demonstrate that there is no increase in NF-κB: DNA association in the lungs of patients who smoke or in the lungs of emphysema patients compared to non-smoking controls. Our clinical data further supports the theory that NF-κB pathway activation is not central to the pathogenesis of COPD. An explanation for the differing results found in the literature regarding NF-4444B pathway activity in the COPD lung may depend on a variety of factors such as what type of sample was taken, whether the patient still smokes, any infections present in the airways, the severity of disease and whether any medication was used.

In summary, we have shown that NF-κB pathway activation is not increased in acute and sub-chronic CS driven murine models of airway inflammation. We have demonstrated that ablation of the upstream NF-κB signalling kinase, IKK-2, in cells of myeloid lineage and in cells of the airway epithelium has no impact on CS-driven lung inflammation. Systemic inhibition of this kinase also failed to impact on the airway inflammation. Together this data package suggests that the inflammation observed after cigarette smoke exposure is not driven through activation of the IKK-2/NF-κB signalling pathway and this may help explain why steroids are ineffective in COPD. We were also able to translate our preclinical data into a clinical setting by demonstrating that there was no change in NF-κB: DNA association between non-smoking patients, smoking patients and emphysema patients. To conclude, this data suggests that IKK-2/NF-κB pathway activation is not central to CS-induced lung inflammation in mice or the pathogenesis of COPD. The identification of NF-κB-independent signalling mechanisms involved in CS-induced lung inflammation may therefore be pivotal in the search for novel therapeutic targets for COPD.

## Supporting Information

Figure S1 **Temporal characterisation of airway inflammation after LPS or 3/14 days of CS challenge.** Mice were challenged with LPS (1 mg/ml) or endotoxin free saline for 30 minutes. Samples were collected at increasing time points after challenge. Figures A, B and C represent BALF levels of TNFα, KC and IL-6, respectively Mice were challenged for 3 or 14 days with CS (500 ml/min, 1 hour, twice daily) or ambient air. Samples were collected at increasing time points after the final challenge. Figures D and E represent BALF levels of KC and IL-23 after 3 days of CS challenge, respectively. Figures F and G represent BALF levels of KC and IL-23 after 3 days of CS challenge, respectively. Data are presented as mean ± s.e.m. of n = 8 observations.
(TIF)

Figure S2 **Characterisation of the IKK-2^ΔEpi^ mice.** Wild type or genetically modified mice were challenged with saline or LPS and the BALF harvested 2 hours later. TNFα and KC levels are represented in Figures A and B, respectively. Data are presented as mean ± s.e.m. of n = 8 observations. # indicates a statistically significant difference (p<0.05) from saline challenged control groups (Mann-Whitney test). * indicates statistical significance (p<0.05) from LPS treated control groups by Kruskal-Wallis one-way-ANOVA with Dunn's multiple comparison post-hoc test.
(TIF)

Figure S3 **Characterisation of a clinically relevant glucocortoid, budesonide and two structurally distinct IKK-2 inhibitors GSK 657311A and TPCA-1 in LPS-induced airway inflammation.** Vehicle or compound was orally dosed to the mice one hour prior to the LPS challenge. BALF samples were collected 2 hours after the LPS challenge. Figures A, B and C represent the levels of KC, TNFα and IL-6, respectively, in the BALF after TPCA1 treatment. Figures D, E and F represent the levels of KC, TNFα and IL-6, respectively, in the BALF after GSK 657311A treatment. Figures G, H and I represent the levels of KC, TNFα and IL-6, respectively, in the BALF after budesonide treatment. Data are presented as mean ± s.e.m. of n = 6–8 observations. # indicates a statistically significant difference (p<0.05) from control challenged groups (Mann-Whitney test). * indicates statistical significance (p<0.05) from LPS challenged vehicle dosed groups by Kruskal-Wallis one-way-ANOVA with Dunn's multiple comparison post-hoc test.
(TIF)
