# Correction: Cigarette Smoke Induced Airway Inflammation Is Independent of NF-κB Signalling

**DOI:** 10.1371/annotation/754d7b19-2dac-479b-a23c-db9fed0431be

**Published:** 2013-10-16

**Authors:** Joseph M. D. Rastrick, Christopher S. Stevenson, Suffwan Eltom, Megan Grace, Meirion Davies, Iain Kilty, Steven M. Evans, Manolis Pasparakis, Matthew C. Catley, Toby Lawrence, Ian M. Adcock, Maria G. Belvisi, Mark A. Birrell

The running title in the PDF is missing a κ. The correct running title is: Role of NF-κB in Smoke Driven Inflammation

All instances of NF-4444B in the text and Reference 8 should appear as NF-κB. IL-1444 in the Discussion section should appear as IL-1β. All instances of ul throughout the text should appear as μl.

In the first paragraph of the Methods section, "Immunohistochemical staining of NF-kB(p65) in IKK-2^ΔEpi^ mice," μM should be μm.

