# Supplementary figures and images for: Cigarette Smoke Induced Airway Inflammation Is Independent of NF-κB Signalling
Source: PLoS One. 2013 Jan 22;8(1):e54128. doi: 10.1371/journal.pone.0054128 (PMC3551940; doi:10.1371/journal.pone.0054128)

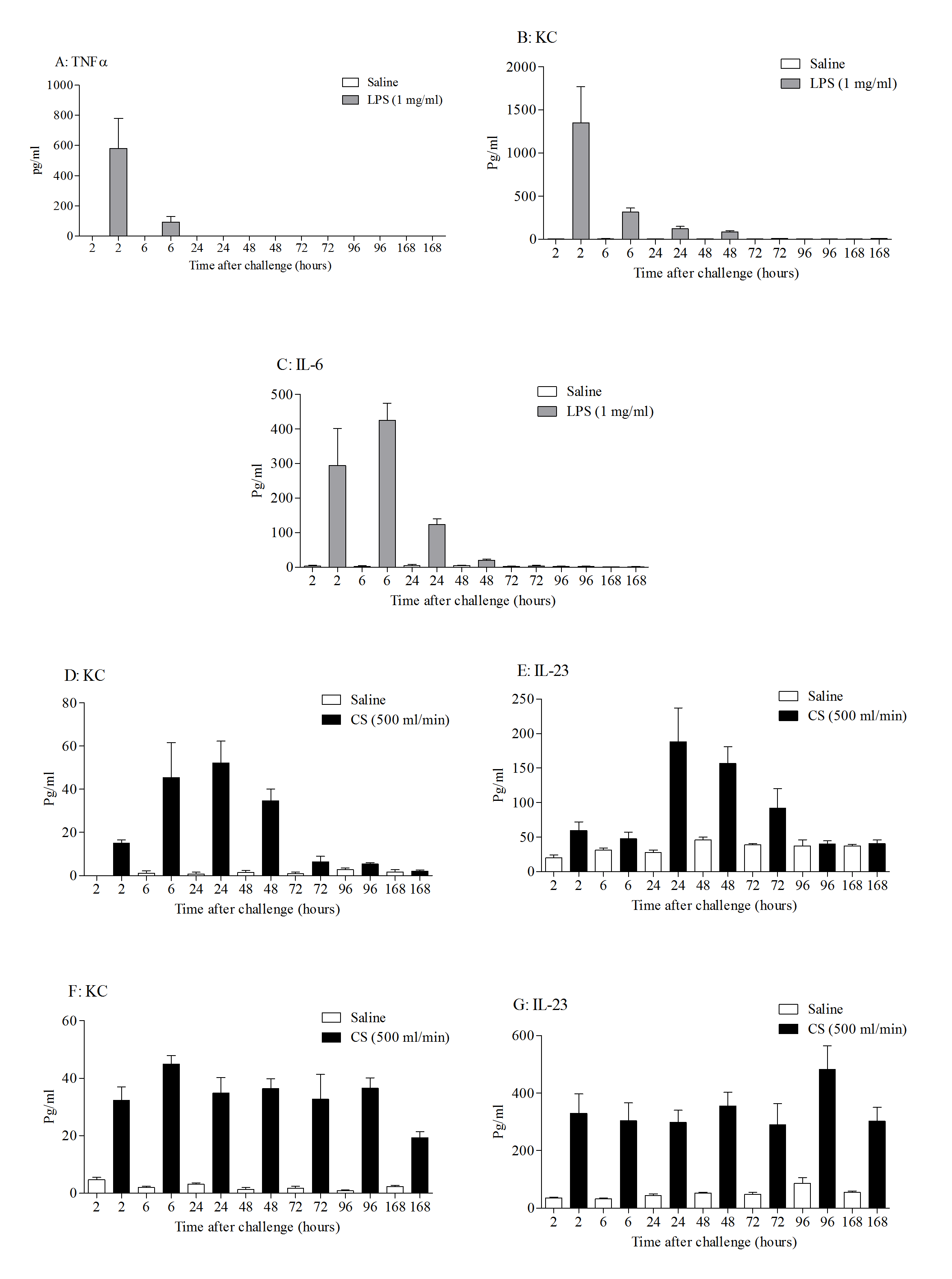

Supplement: Figure S1 — Temporal characterisation of airway inflammation after LPS or 3/14 days of CS challenge. Mice were challenged with LPS (1 mg/ml) or endotoxin free saline for 30 minutes. Samples were collected at increasing time points after challenge. Figures A, B and C represent BALF levels of TNFα, KC and IL-6, respectively Mice were challenged for 3 or 14 days with CS (500 ml/min, 1 hour, twice daily) or ambient air. Samples were collected at increasing time points after the final challenge. Figures D and E represent BALF levels of KC and IL-23 after 3 days of CS challenge, respectively. Figures F and G represent BALF levels of KC and IL-23 after 3 days of CS challenge, respectively. Data are presented as mean ± s.e.m. of n = 8 observations. (TIF) [file pone.0054128.s001.tif]

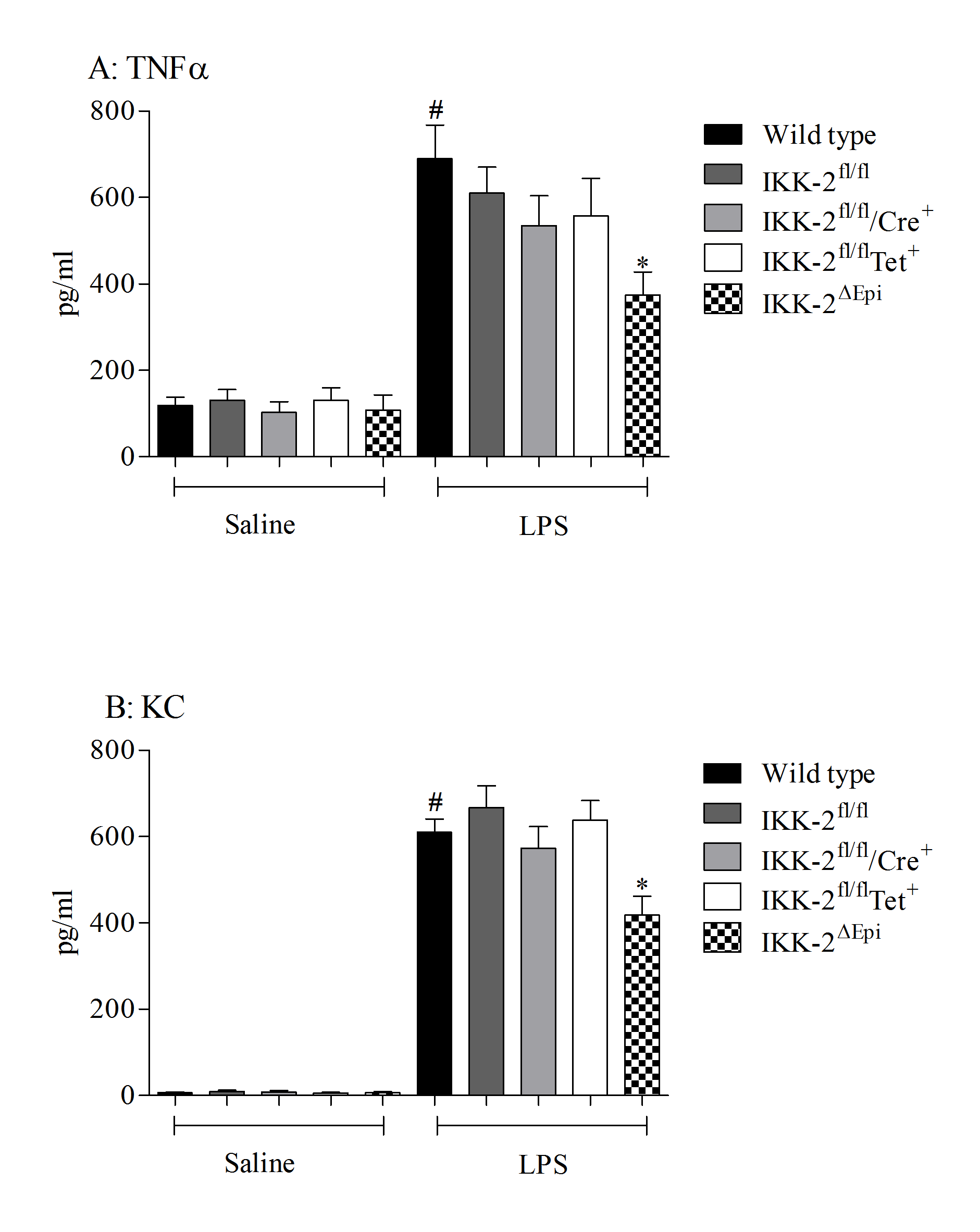

Supplement: Figure S2 — Characterisation of the IKK-2ΔEpi mice. Wild type or genetically modified mice were challenged with saline or LPS and the BALF harvested 2 hours later. TNFα and KC levels are represented in Figures A and B, respectively. Data are presented as mean ± s.e.m. of n = 8 observations. # indicates a statistically significant difference (p<0.05) from saline challenged control groups (Mann-Whitney test). * indicates statistical significance (p<0.05) from LPS treated control groups by Kruskal-Wallis one-way-ANOVA with Dunn's multiple comparison post-hoc test. (TIF) [file pone.0054128.s002.tif]

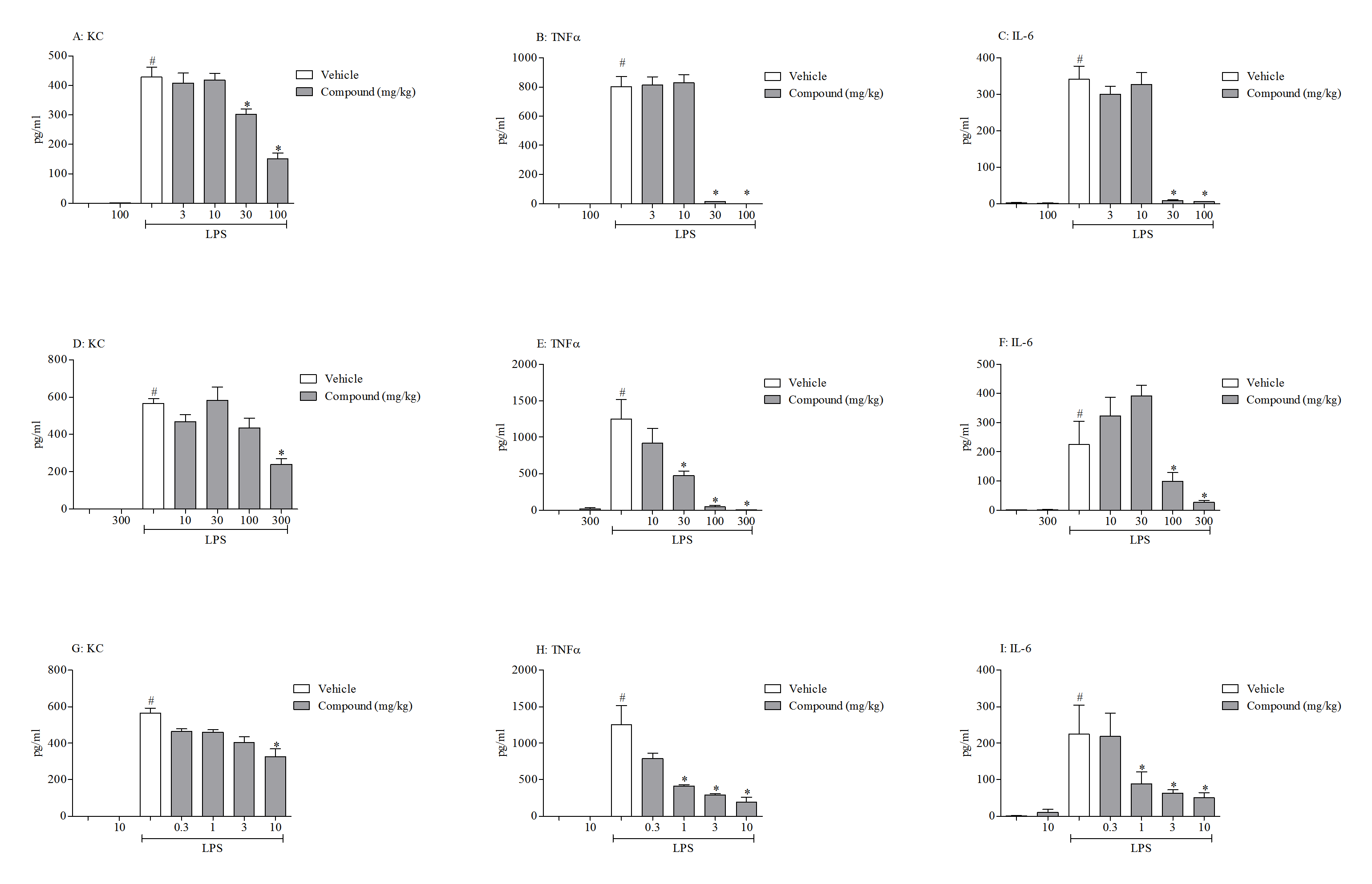

Supplement: Figure S3 — Characterisation of a clinically relevant glucocortoid, budesonide and two structurally distinct IKK-2 inhibitors GSK 657311A and TPCA-1 in LPS-induced airway inflammation. Vehicle or compound was orally dosed to the mice one hour prior to the LPS challenge. BALF samples were collected 2 hours after the LPS challenge. Figures A, B and C represent the levels of KC, TNFα and IL-6, respectively, in the BALF after TPCA1 treatment. Figures D, E and F represent the levels of KC, TNFα and IL-6, respectively, in the BALF after GSK 657311A treatment. Figures G, H and I represent the levels of KC, TNFα and IL-6, respectively, in the BALF after budesonide treatment. Data are presented as mean ± s.e.m. of n = 6–8 observations. # indicates a statistically significant difference (p<0.05) from control challenged groups (Mann-Whitney test). * indicates statistical significance (p<0.05) from LPS challenged vehicle dosed groups by Kruskal-Wallis one-way-ANOVA with Dunn's multiple comparison post-hoc test. (TIF) [file pone.0054128.s003.tif]
